# Performance Evaluation of Proximal Sensors for Soil Assessment in Smallholder Farms in Embu County, Kenya

**DOI:** 10.3390/s16111950

**Published:** 2016-11-19

**Authors:** Kristin Piikki, Mats Söderström, Jan Eriksson, Jamleck Muturi John, Patrick Ireri Muthee, Johanna Wetterlind, Eric Lund

**Affiliations:** 1 Regional Office for Africa, International Center for Tropical Agriculture (CIAT), Kasarani Rd., ICIPE Complex, P.O. Box 823-00621, Nairobi, Kenya; mats.soderstrom@slu.se; 2 Department of Soil and Environment, Precision Agriculture and Pedometrics, Swedish University of Agricultural Sciences (SLU), Box 234, SE-53223 Skara, Sweden; johanna.wetterlind@slu.se; 3 Department of Soil and Environment, Biogeochemistry, Swedish University of Agricultural Sciences (SLU), Box 7014, SE-75007 Uppsala, Sweden; jan.o.eriksson@slu.se; 4 School of Agriculture, University of Embu (UoEm), P.O. Box 6-60100, Embu, Kenya; jjamkenya@yahoo.com; 5 Ministry of Agriculture, Embu, Kenya; patrickireri66@gmail.com; 6 Veris Technologies Inc., 1925 Clay Ridge Ct., Salina, KS 67401, USA; lunde@veristech.com

**Keywords:** East Africa, proximal sensor, soil assessment, subsistence farming

## Abstract

Four proximal soil sensors were tested at four smallholder farms in Embu County, Kenya: a portable X-ray fluorescence sensor (PXRF), a mobile phone application for soil color determination by photography, a dual-depth electromagnetic induction (EMI) sensor, and a LED-based soil optical reflectance sensor. Measurements were made at 32–43 locations at each site. Topsoil samples were analyzed for plant-available nutrients (N, P, K, Mg, Ca, S, B, Mn, Zn, Cu, and Fe), pH, total nitrogen (TN) and total carbon (TC), soil texture, cation exchange capacity (CEC), and exchangeable aluminum (Al). Multivariate prediction models of each of the lab-analyzed soil properties were parameterized for 576 sensor-variable combinations. Prediction models for K, N, Ca and S, B, Zn, Mn, Fe, TC, Al, and CEC met the setup criteria for functional, robust, and accurate models. The PXRF sensor was the sensor most often included in successful models. We concluded that the combination of a PXRF and a portable soil reflectance sensor is a promising combination of handheld soil sensors for the development of in situ soil assessments as a field-based alternative or complement to laboratory measurements.

## 1. Background

### 1.1. Soil Testing Is Important

Agriculture is the backbone and livelihood of a majority of the population in East Africa. Economic and efficient farm production is highly determined by soil conditions. Soil testing is an important procedure to undertake, since it determines the fertility status, i.e., the physical and chemical properties that affect a soil’s suitability for growing plants. Soil health determines how well the soil can function [1]. Different methods of soil assessment have been adopted over the years. An alternative or complement to laboratory soil analyses could be to test the soil directly in the field, e.g., by using handheld instruments. Knowledge of the local soil properties is crucial to farmers as it enables them to determine best management practices. The main bottlenecks for soil testing among smallholders in this area are (1) lack of knowledge on why soil testing is important; (2) inadequate laboratory facilities and inadequate qualified staff to do the lab work; and (3) expensive cost for lab analyses in relation to a farmer’s budget. The most important soil properties to analyze are pH and soil nutrient status, as well as soil texture.

### 1.2. Continental Digital Soil Maps

There are two recently published digital soil maps of quantitative soil properties related to soil fertility, one covering the African continent except desert areas [2] and the other covering Africa south of the Sahara [3]. In spite of the relatively high resolution (250 m and 500 m, respectively), such large-extent maps cannot be assumed to be directly applicable at farm or regional scale without prior local validation and/or adaptation [4]. These kinds of soil maps can be useful at the regional level, for example, as a guide for fertilizer retailers on which fertilizer blend types to keep in stock. It can also be used in discussions in knowledge groups of farmers and extension officers. For accurate information on the soil fertility status on a given farm, local measurements are still needed.

### 1.3. Proximal Soil Sensing

An alternative or a complement to laboratory analyses could be to test the soil directly on the farm, using mobile proximal sensors. Viscarra Rossel et al. [5] define proximal soil sensing as measurement of signals from the soil with field-based sensors held in contact with or close to (<2 m) the soil. Measurement in situ has potential to be cost- and labor-effective compared to laboratory analyses but it is a challenge to make accurate predictions under the non-standardized soil conditions prevailing on the farm (varying soil moisture and soil structure). A multitude of research studies has dealt with the issue of calibrating the often indirect field-based measurements against lab analyses to produce accurate values of a variety of soil properties [6]: electromagnetic conductivity (ECa) has been used widely to map spatial variation patterns of agricultural fields but also for prediction of specific soil properties, for example, cation exchange capacity (CEC), soil moisture content, soil organic carbon (SOC) content, and the fractions of clay, silt, and sand [7]; element concentrations measured with a portable X-ray fluorescence sensor (PXRF) in situ have been demonstrated to correlate with soil texture, SOC, and CEC [8,9,10]; Jung et al. [11] showed that a multispectral camera can be used to predict SOC; and, recently, even the built-in cameras in mobile phones have been used as proximal soil sensors—Aitkenhead et al. [12] predicted SOC from the registered values of soil color (red, green, and blue bands) registered with a mobile phone camera.

There is, however, a scarcity of studies of relevance for the application of proximal sensing technologies in smallholder farming systems in Africa. Firstly, tropical soils are underrepresented in the proximal soil sensing literature and, secondly, the fact that the fields are smaller and the landscape is more heterogeneous than in many other investigated agricultural systems calls for other calibration strategies; with the small farm size, there is little use in making field-specific sensor calibrations. Regional (e.g., county) calibrations are needed for proximal sensing to be a viable option in smallholder landscapes.

### 1.4. Overall Goal and Specific Research Questions

The overall aim of this work was to screen a number of proximal sensors to find out which technique can be used most effectively in assessments of soil properties in smallholder farming systems in East Africa. We see screening as a delicate task that is the first step in a longer process and it must be followed up by more in-depth work towards a working sensor-based strategy for farm scale soil assessment. The work aimed at answering the following research questions:

1. For which lab-analyzed soil properties could functional, robust, and accurate sensor-based prediction models be calibrated?
2. How should validation be designed, if one wants to identify spatially robust models?
3. How is the variation in soil properties within and between sites translated to management advice given to farmers?

From the answers to these questions we were able to draw conclusions on which sensors were useful and how further method development should proceed. By functional we mean that the instrument should work in the investigated environment. If concentrations of a nutrient estimated by a sensor in most cases are below the detection limit, this sensor is not functional for that specific nutrient. By robust we mean that a calibrated model should be applicable to other sites in the county than those where the calibration soil samples were taken. By accurate we mean that the error should be so small that the soil property predictions can safely be used as a basis for management advice/decisions.

## 2. Materials & Methods

### The Study Area

**Figure 1:**
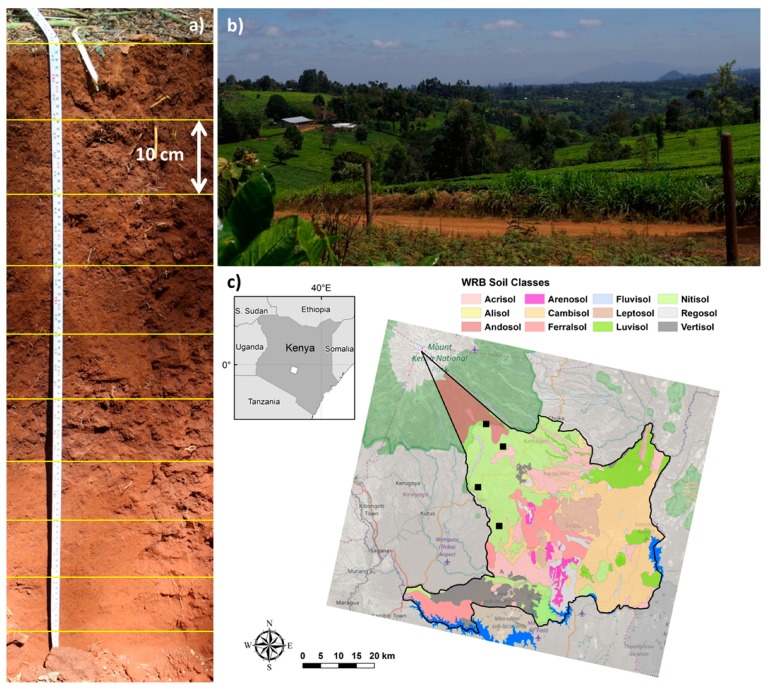
The soils and landscape of Embu County: (**a**) a soil profile from the nitisol region of Embu County; (**b**) a landscape photo from the tea growing area; and (**c**) World Reference Base (WRB) soil classes [13] of Embu County at Mt. Kenya according to Batjes et al. [14]. The study sites are marked with black squares. The background map is from OpenStreetMap (© OpenStreetMap contributors, data licenced on terms of ODbL 1.0, cartography licensed as CC BY-SA).

The study was conducted on the southern slopes of Mt Kenya in Embu County in Eastern Kenya (Figure 1). Four sites along an altitudinal transect of the southwestern slopes of Mt Kenya were selected. About 70% of the inhabitants of Embu County, and almost 90% of the households, are engaged in agricultural activities. The northern part of the county relies mainly on cash crops such as coffee (*Coffea arabica* L.) and tea (*Camellia sinensis* L.), while the southern part mainly produces food crops such as maize (*Zea mays*), beans (*Phaeolus vulgaris* L.), fruits, and vegetables. The largest proportion of arable land in the county is used for agriculture, with farms averaging a little less than one hectare due to land fragmentation over the years. The total acreage under cash crops is 19,000 ha.

## 3. Sensors and Sampling Design

The sensors tested were: a portable X-ray-fluorescence (PXRF) sensor, a handheld optical sensor based on two specific wavelengths, a mobile phone application for soil color determination by photography (soil color app), and an electromagnetic induction (EMI) sensor.

**Figure 2:**
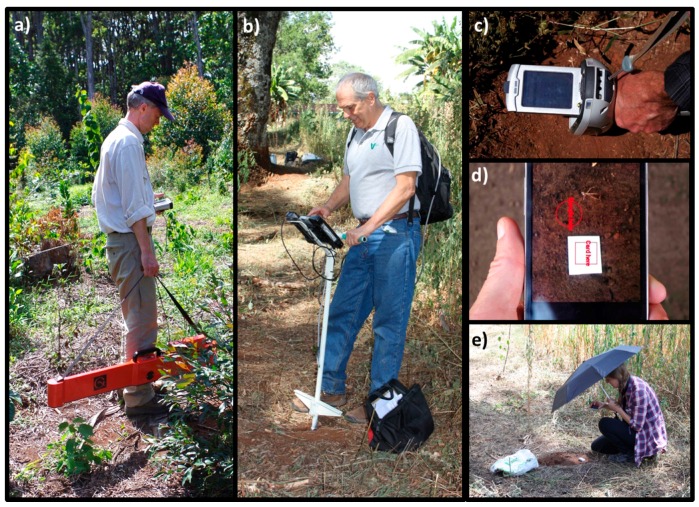
The proximal sensors tested: (**a**) an electromagnetic induction sensor; (**b**) an optical sensor; (**c**) a portable X-ray fluorescence sensor (PXRF); (**d**) a smartphone soil color application; and (**e**) the umbrella used for shading.

The EMI sensor was an EM38-MK-2 instrument manufactured by Geonics Ltd., Mississauga, ON, Canada. Such instruments have proved useful for mapping a range of soil properties that affect the apparent soil electrical conductivity (ECa) [15]. This particular equipment has two receiver coils, which means that it measures over two soil depths simultaneously. The receivers are located 0.5 m and 1.0 m from the transmitter coil and, with the instrument held at 0.15 m above ground (see Figure 2a), the effective measurement depths for ECa are about 0.5 m and 1.0 m. The instrument also measures magnetic susceptibility (MSa), which has been demonstrated to be a useful predictor of CEC, SOC content, and depth of the A horizon in some tropical soils [16]. The measurement depths for MSa are shallower than for ECa (about 0.25 and 0.5 m).

The optical sensor was a handheld (prototype) version of the Optic Mapper (Figure 2b, Veris Technologies Inc., Salina, KS, USA). It measures reflectance at specific wavelengths (bandwidth 20 nm), in the red (R, 660 nm) and the near-infrared (IR, 940 nm) regions of the electromagnetic spectrum. The two wavelengths were chosen for their predictive power of SOC. The sensor development and initial performance testing has been described in detail by [17]. In this study we used R, IR, and their quotient (R/IR) as predictor variables in the predictive modeling of different soil properties.

**Figure 3:**
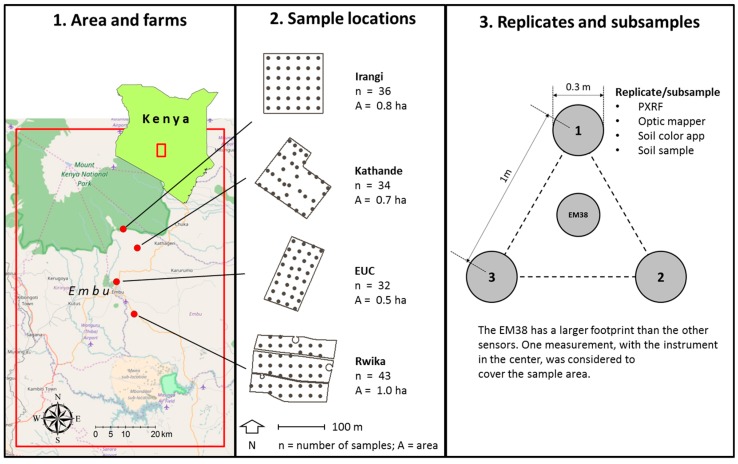
Sampling location and design. The map to the left shows where the four sites are located in Embu County, Kenya. The center panel shows where the samples were taken in each of the four sites and the panel to the right shows the geometry of replicates at the sample location. Subsamples are replicates. EUC = Embu University College. The background map is from OpenStreetMap (© OpenStreetMap contributors, data licenced on terms of ODbL 1.0, cartography licensed as CC BY-SA).

A PXRF sensor (Figure 2c, we used a Niton XL3t GOLDD+; Themo Scientific, Billerica, MA, USA) is capable of detecting abundancies of a range of elements, from magnesium and heavier. The functionality of PXRF has been described e.g., in [18]. In this study, we used the PXRF data for predictions of soil properties. Three measurements were done at each soil sample location in the field (Figure 3), and the average of the element concentrations was used. Before the measurement, the layer of plant litter (10–20 mm) was removed and the soil was slightly trampled to make the surface smooth and compact. Measurement time was four minutes in total for each PXRF measurement, one minute per filter (filters in this case are mechanisms built into the instrument that allows the X-ray energy to be modified to preferentially enhance the analysis of certain elements), i.e., twelve minutes per sampling site. The responsive volume of soil is very small when using PXRF; the penetration depth is only a few mm and the area with the instrument used is about 1 cm^2^. One of the fields was a tea plantation and the soil surface was covered by lots of plant litter and roots, which prohibited meaningful data collection directly on the ground. There, PXRF measurements were done instead in the field, but on augered soil collected for soil chemical analyses. A measurement was considered to be above the limit of detection if it was larger than three times the returned standard deviation (as suggested by e.g., Weindorff et al. [19]).

The soil color app was developed by Noisy Flowers LLC for the Land-Potential Knowledge System (LandPKS [20]) with support from the United States Agency for International Development (USAID) and the United States Department of Agriculture, Agricultural Research Service (USDA-ARS). It uses the three bands of the RGB images obtained by the smartphone camera. A white TiO_2_ reference card was used to correct for variation in the ambient light (Figure 2d). A black umbrella was used for shading from direct sunlight during the measurements (Figure 2e). The output data from the app was reference-corrected values for red (R), green (G), and blue (B) bands, as well as the color code according to Munsell (see, e.g., [21]). The app was installed in a Samsung Android S4 mini GT-191 with a CMOS, 8 MP sensor. In addition to using the app outdoors, it was used in the office, where the light environment was not as variable. The soil sample bags were opened and one measurement was made in each bag.

## 4. Field Measurements, Sampling, and Lab Analyses

Three replicate measurements were made with the PXRF, the optic mapper, and the soil color app at 32–43 locations in an approximate regular grid at each of the four sites (Figure 3). The EMI sensor has a larger footprint (measured area) than the other sensors, and one measurement with the instrument placed in the center was considered to represent the entire sampling area. Two soil cores from the 0–20 cm were taken at each of the three locations where the sensor measurements were replicated. The soil sampling locations were positioned with a TDS Nomad GPS (Tripod Data Systems, Corvallis, OR, USA). The approximate accuracy of the positioning was ±3–5 m). All spatial data were projected onto the planar reference system WGS 84/UTM zone 37S (EPSG projection 32737). For the purpose of site description, two pits were made at each farm (site) and soil samples were taken at several depths.

The soil samples were air-dried at 30 °C and sieved to pass 2 mm after larger peds had been crushed to smaller pieces. Soil pH was measured in a soil:water ratio of 1:2.5; total carbon and nitrogen were measured after dry combustion using an Elementar Vario max cube (ISO 10694, first edition 1995-03-01); P, K, Mg, Ca, S, Zn, Mn, Fe, B, Cu, and Mo were extracted in 0.2N CH_3_COOH + 0.2N NH_4_NO_3_ + 0.015N NH_4_F + 0.013N HNO_3_ + 0.001M EDTA (Mehlich-III) [22]; exchangeable Al was extracted in 1 M KCl. The elements were analyzed with ICP-OES (Thermo Dual—6000 series). Contents of sand (2–0.05 mm), silt (0.05–0.002 mm), and clay (<0.002 mm) were determined by sedimentation analysis (hydrometer) using 10% sodium hexametaphosphate as the dispersing agent. No deferration was done before the sedimentation. Melich-III extraction was chosen at all sites, irrespective of pH, in order to get a consistent dataset.

The cation exchange capacity (CEC) (cmol_c_·kg^−1^) was calculated according to Equation (1), where concentrations of exchangeable base cations Ca^2+^, K^+^, Mg^2+^, and Na^+^ (from the Mehlich III extract) were expressed as g·kg^−1^ soil, and concentrations of exchangeable H^+^ and “other cations” (Al^3+^, Fe^2+^, Mn^2+^, etc.) were expressed in % and estimated from soil pH according to procedure by Crop Nutrition Laboratory Services Nairobi, Kenya. The denominators below the base cation symbols are the molar weights divided with the valence of the cation. The CEC of clay was estimated from soil profile data (supplement Tables S1–S4) by dividing CEC for total soil with relative clay content, i.e., assuming that all CEC derives from the clay. This leads to an overestimate due to the contribution to CEC from organic matter, but can give an indirect indication if organic matter content is low. Therefore, data from the deepest horizons were used.

(1) $$\mathrm{CEC}=\left(\frac{\left(\frac{\left[{\mathrm{Ca}}^{2+}\right]}{200}+\frac{\left[K^{+}\right]}{390}+\frac{\left[{\mathrm{Mg}}^{2+}\right]}{120}+\frac{\left[{\mathrm{Na}}^{+}\right]}{230}\right)}{\left(100-\left(\left[H^{+}\right]+\text{other}\;\text{cations}\right)\right)}\right)\times 100$$

All analyses except the determination of particle size distribution and the total contents of C and N were made at Crop Nutrition Laboratory Services Nairobi, Kenya. The latter properties were instead determined at CIAT Laboratory, Ltd., Nairobi, Kenya.

### 4.1. Screening Procedure

**Figure 4:**
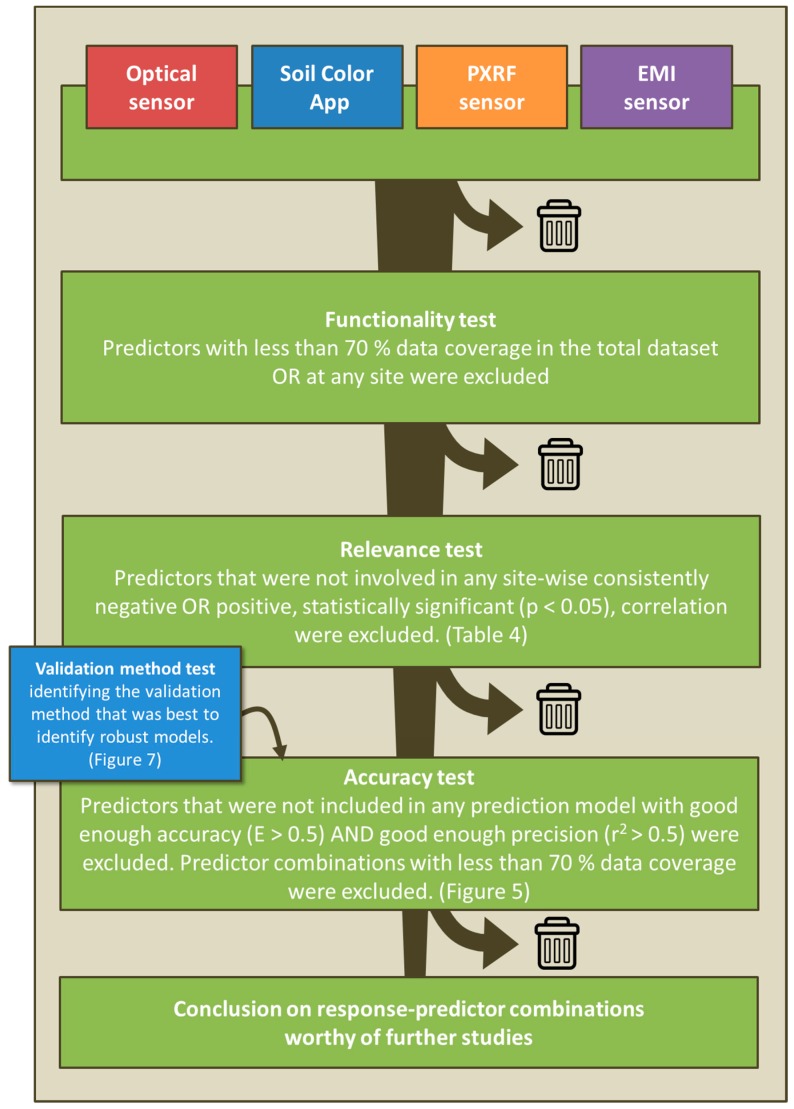
Schematic overview of the screening procedure carried out to select functional, robust, and accurate sensor-based prediction models. PXRF = portable X-ray fluorescence, EMI = electromagnetic induction, E = modelling efficiency, r^2^ = coefficient of determination.

An overview of the screening procedure carried out to sift out functional, robust, and accurate sensor-based prediction models is given in Figure 4. In the first step, the data quality was checked. Frequency distributions of all lab variables and sensor variables were scrutinized and 13 of the total 116,403 values were removed (~0.1‰), as they were suspected to be erroneous.

### 4.2. Functionality Screening

Failure of measurement was a problem for some sensor-measured variables. In the next step, a functionality screening was done to prevent variables that were difficult to measure from being included in the predictive modeling. Sensor variables for which >30% of the measurements at any site, or in the total dataset, were below the detection limit or for any other reason missing were omitted from further analyses.

### 4.3. Relevance Screening

Bringing predictors that have no relation to the response variable into a calibration dataset considerably increases the risk of parameterizing overfitted (non-robust) prediction models [23]. Therefore, the functionality screening was followed by a relevance screening step. Pearson’s correlation coefficients (r) and their probabilities (*p*) were calculated for all sensor–lab variable pairs, for each site, individually. Variables that were not part of any significant pairwise correlation at any site were omitted from further analyses, and so were lab–sensor variable pairs that showed a significant positive correlation at some sites and a significant negative correlation at other sites. Such mixed-sign correlations were considered most likely to be due to chance, rather than to a direct—or indirect—useful, causal relationship. It needs to be pointed out here that this is just a rough and rapid screening. The r values are calculated with no respect to whether any relationship between two variables is actually linear in nature. Neither is any autocorrelation between the samples in a field taken into account. Non-independence among samples implicates a risk that correlations are being judged as significant when they are not (at the specified *p*-value), i.e., the relevance screening will be less strict than it would be if there was no autocorrelation.

### 4.4. Validation Strategy

In order to validate the robustness of a model, i.e., how well it performs on new datasets that have not been used for calibration (or in this case: how well it performs on sites where no calibration soil samples were taken), it is essential to choose an appropriate validation design. Two different designs were tested and compared and the one that worked best to identify robust models was used in the subsequent accuracy screening. The two tested validation methods were: (1) a random threefold cross-validation and (2) a leave-one-site-out cross-validation. In the random threefold strategy, a random third of the soil samples were omitted from the calibration set and in the leave-one-site-out calibration, all samples from one site were left out. Models were calibrated based on the remaining samples and applied on the left-out samples. This was repeated until all samples had been left out once, and there was a full dataset with both predicted and measured values for all samples. In the random threefold validation, the same random split into three datasets was done once and used for all response variables. Two validation measures were computed: (1) the modelling efficiency (E; [24]) and (2) the coefficient of determination of a linear regression between predicted and measured values (r^2^). The E value is a measure of how well a model performs and can be calculated for a set of predictions where corresponding measured reference values are available. E takes values between −∞ and 1, where E = 1 indicates a perfect fit, E = 0 means that the models predictions are just as accurate as the mean of the reference values and E > 0 indicates a very poor model.

### 4.5. Accuracy Screening

Prediction models were calibrated with the sensor variables as predictors and the lab variables as responses. We chose to use multivariate adaptive regression splines models (MARSplines; [23]) because they are flexible, yet less prone to overfitting than some alternative models (based on our experience). A MARSplines model consists of piecewise linear regression models (basis functions), which are valid within defined intervals of the X variables (the predictors). MARSplines models can be parameterized with interactions among the basis functions but we chose to calibrate simple additive models, to reduce the risk of overfitting. The Earth package [25] in the R software ([26]; version 3.1.2) was used for the parameterization and deployment of MARSplines models. All possible combinations of 1–3 predictor variables (=576 predictor combinations) chosen from the full set of soil properties measured by the five sensors were tested for each response variable. An E > 0.5 and an r^2^ > 0.5 were used as accuracy criteria for a model worth examining further in coming studies (i.e., the screening criteria of the study).

### 4.6. Daktari-wa-Udongo

It is interesting to explore the variability of soil properties, but for the farmers it is not very useful until the measured soil properties have been translated into management advice. We therefore used the *Daktari-wa-Udongu* (Soil doctor) advice of the CropNuts lab (Nairobi, Kenya) to derive management advice for each sample. This service is used in East Africa for selection of, e.g., fertilizer and liming recommendations based on soil analyses, crops grown, and expected yields. The advice was based on the following crops and yield levels: Irangi, 8.5 tons of tea per hectare; Kathande, 7 tons of tea per hectare; EUC, 5 tons of maize per hectare; and Rwika, 6.3 tons of maize per hectare.

## 5. Results

### 5.1. Soil Properties

The soils in the study areas are clayey with a clay content of the topsoil of around 40% at three of the sites and 59% on average at EUC (Table 1). At the lowest site, Rwika, topsoil C was 2.1% on average, increasing with altitude up to 7.4% at Kathande, and then lower again at Irangi. The soils at higher altitude are rather acid, around pH 4.6 at Irangi and pH 4.2 at Kathande. The soils at the two other sites are more than one unit higher in pH. A generally low pH, especially at higher altitudes, has also been recorded in previous studies (data from AfsoilGrids250; [2]). Cation exchange capacity varied from 18.0 cmol_c_·kg^−1^ at EUC to 2.4 cmol_c_·kg^−1^ at Irangi. The low CEC at Irangi, despite a high TC content, seemed to be due to a very low CEC of the clay, indicating a more strongly weathered soil with dominance of sesquioxides in the clay fraction. The Kathande soil also had a very low CEC of the clay, while the two soils at lower altitude had higher values, indicating less oxides in the clay fraction.

Inferred from the SOTER map [14] (Figure 1), nitisols are the dominating soil in the study area. According to IUSS Working Group WRB [13], the Reference Soil Group of the Nitisols accommodates deep, well-drained, red tropical soils with diffuse horizon boundaries. Nitisols are strongly weathered soils, but far more productive than most other tropical soils. From the soil profiles of the two soil pits at each site, it was, however, difficult to detect the shiny ped faces characteristic of the B-horizon in Nitisols [13]. Texture data on the soil profiles showed an increased clay content in the upper part of the B-horizon (See Tables S1–S4 in supplementary material), but it did seldom increase more than 20% relative over 15 cm depth, as required for Nitisols. The CEC of the clay and sum of exchangeable bases (Mehlich-III) and exchangeable Al were below 16 and 12 cmol_c_·kg^−1^, respectively, indicative of a Ferralic horizon. To resolve whether these soils are Ferralsols or Nitisols, data on water-dispersible clay and dithionite and oxalate-extractable iron are needed.

Descriptive statistics of the measured soil properties are given in Table 1. It can be noted that there is often, but not always, a relatively small variation within the sites and a large difference between sites. This is not surprising, considering the relatively small sampled area at each site and the fact that the sites are distributed along an altitudinal transect (of Mt. Kenya). It can also be noted that the Zn content at the EUC site deviated substantially from the other sites, and so did the Cu content in Kathande. In Table 2, the site-wise correlations between all pairs of the lab-measured soil properties are summarized. Many of the soil properties were positively correlated, but the clay content was negatively correlated to N, P, and K. As expected, there was a negative correlation between exchangeable Al and soil pH at two sites.

### 5.2. Screening Results

#### 5.2.1. Functionality Screening

The following elements measured with the PXRF were omitted from the predictive modelling because they were frequently below the detection limit (at >30% of the measured locations at any site or in the total dataset): aluminum (Al), antimony (Sb), arsenic (As), barium (Ba), bismuth (Bi), cadmium (Cd), calcium (Ca), cesium (Cs), chlorine (Cl), cobalt (Co), gold (Au), hafnium (Hf), lead (Pd), magnesium, (Mg), mercury (Hg), niobium (Nb), phosphorous (P), rhenium (Re), scandium (Sc), selenium (Se), silica (Si), silver (Ag), sulfur (S), tallium, (Ta), thorium (Th), tin (Sn), uranium (U), and tungsten (W). The shallow MSa readings from the EMI sensor had more often than not negative values and this variable was therefore also discarded in this screening step.

#### 5.2.2. Relevance Screening

The following variables were removed because they did not show any significant correlation (*p* < 0.05; disregarding any spatial autocorrelation) with any lab-variable at any site: the G and the B bands from the soil color app (both indoor and outdoor), the remaining three EMI sensor variables (shallow ECa, deep ECa, and deep MSa) and the PXRF sensor measurements of copper (Cu), nickel (Ni), and vanadium (V). A summary of the signs of the correlations that were significant at *p* < 0.05 at least at one of the four sites is given in Table 3. It was the sensor variables presented in this table that were passed on to the predictive modeling.

#### 5.2.3. Accuracy Screening

**Figure 5:**
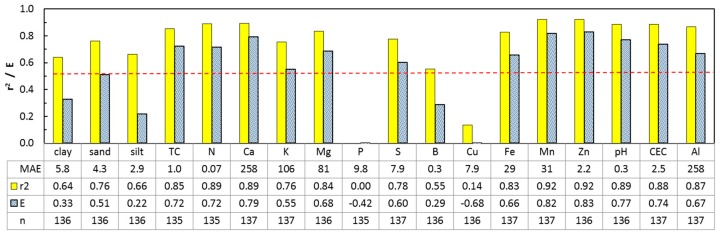
Results from the leave-one-site-out validation. The bars show E and r^2^-values for one of the five best models for each response variable (included predictors indicated by a star in Table 4). The dotted bars show the modeling efficiency. The plain filled bars show the coefficient of determination for a linear regression between predicted and measured values. TC = total carbon, TN = nitrogen, and CEC = cation exchange capacity. The red line indicates r^2^ = 0.5 or E = 0.5 (the screening criteria for relevance and accuracy). MAE = mean absolute error. The units of the MAEs are found in Table 1.

The results from the leave-one-site-out validation are presented in Figure 5. The bars show E and r^2^-values for the best model for each response variable (included predictors indicated by a star in Table 4). The red hatched line shows the threshold used for judging whether models are interesting enough to be tested further (E = 0.5 and r^2^ = 0.5). The prediction models for N, Mg, Ca, S, Zn, Mn, Fe, TC, pH, and CEC had E and r^2^ values exceeding these thresholds, while P, B, Cu, and the three texture variables could not be modeled with satisfactory accuracy.

### 5.3. Comparison of Validation Methods

**Figure 6:**
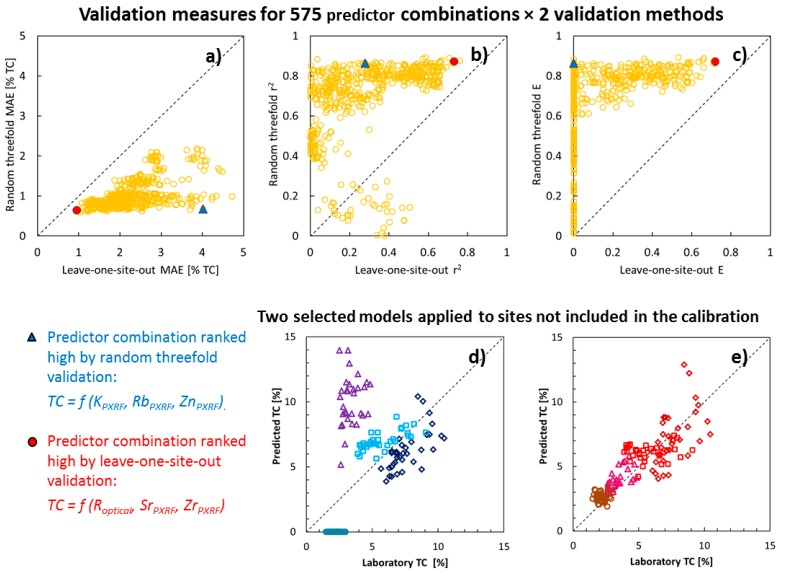
Comparison of validation methods. In the upper row of plots (**a**–**c**), each point represents the validation of one prediction model for total carbon (TC). There are 575 points per plot, each representing a unique predictor combination. The *y*-axes show results from the random threefold validation, while the *x*-axes show results from the leave-one-site-out validation. For two of the predictor combinations, the best in the leave-one-site-out validation (red-filled circle marker) and the best in the random threefold validation (blue-filled triangle marker), predicted values are plotted against measured values (leave-one-site-out method) in plots (**d**,**e**). The different shapes of the symbols in (**d**,**e**) indicate different sites. MAE = mean absolute error, r^2^ = the coefficient of determination for a linear regression between the predicted and the measured value. Some values were out of the range for the plots and values <0 were set to 0.

In Figure 6, the leave-one-site-out validation and the random threefold cross-validation are compared. In the three plots in the upper row, the validation measures (MAE, r^2^, and E) for the random threefold validation is plotted against the same validation measure calculated for the leave-one-site-out validation. Each point represents one predictor combination, and the response variable in this example was TC. Two predictor combinations were studied in more detail; the best predictor combination based on the leave-one-site-out validation method [R_optical_, Sr, Zr; Table 4] is marked by filled red symbols, and marked with blue symbols is the best predictor combination [K, Rb, Zn] based on the random threefold validation. From studying the scatterplots, it is evident that a predictor combination rendering a good validation result by the random threefold validation method does not necessarily show good validation measures in the leave-one-site-out validation. In other words, there is a considerable risk that the random threefold validation indicates that a combination of sensor variables has a high predictive power, while, in fact, predictions at new sites (i.e., sites where no calibration samples were collected) are really poor. One very clear example of this is the TC predictions for the Rwika site: all predictions were negative (shown as 0 in the plot), even though the predictor combination was judged as one of the best from the random threefold validation.

### 5.4. Spatial Variation Structure in Soil Properties and Soil Test-Based Advice

**Figure 7:**
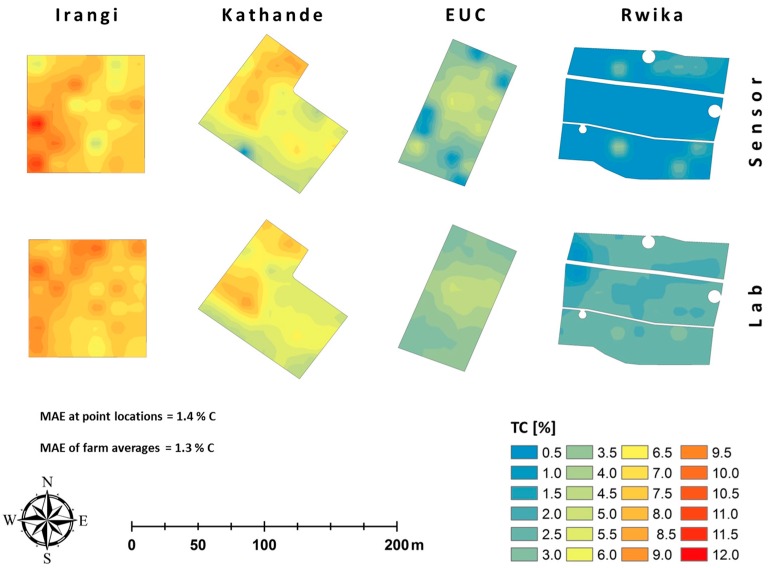
Variation in total carbon (TC) within and between fields. The point location values have been interpolated by inverse squared distance weighting to a 5 m × 5 m grid and are visualized by a bilinear smoothing spline. The upper maps are based on TC values predicted by the sensor-based model, while the lower maps were based on lab-measured values. Descriptive statistics for each site can be found in Table 1. The legend, the scale bar, and the north arrow are valid for all maps. MAE = mean absolute error.

In Figure 7, maps based on measured and predicted TC values (the predictors marked with a star in Table 4) are presented for the four sites. There was a considerable variation in TC between sites that followed the altitudinal transect (higher TC at higher elevations). This was well reproduced by the sensor-based predictions. There was also variation in TC within the sites. From visual judgement, major variation patterns in TC at EUC and Kathande seem to be largely similar in the sensor-based and the lab-based maps. At Irangi and Rwika any similarity was less obvious.

**Figure 8:**
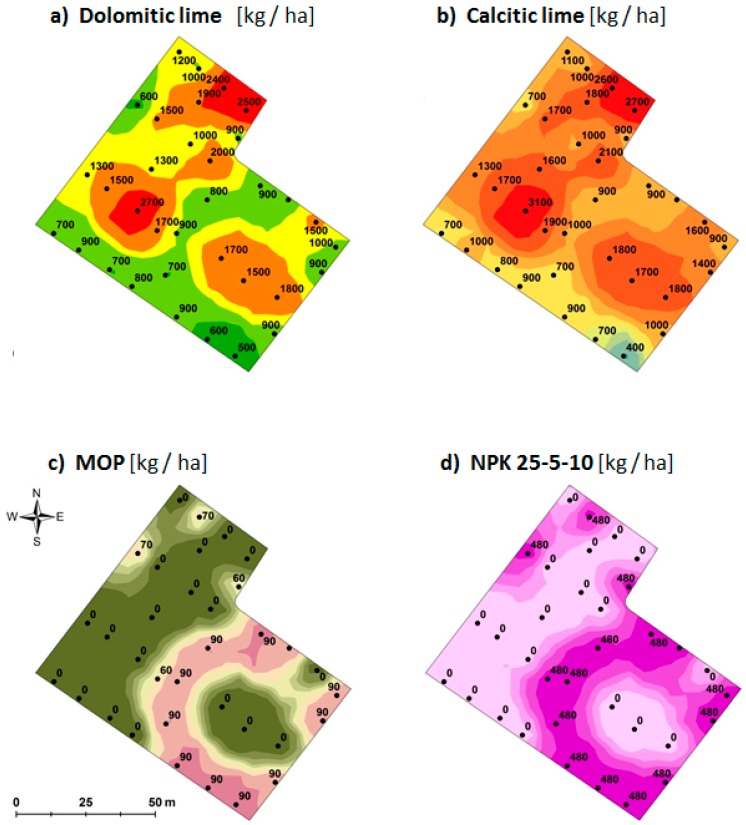
Spatial variation in input recommendations on the Kathande tea site. Because of the low pH (Table 1), pH correction by dolomitic and calcitic lime (**a**,**b**) was recommended. On this soil, there will be little response to inorganic fertilizer until lime is applied to correct pH and reduce aluminium levels. Recommended topdressing fertilizers are shown in (**c**,**d**). MOP (Muriate of Potash) = KCl. The recommendations are for a yield level of 7 tons tea per hectare. The calcitic lime should have a Ca content of 35%–40% and a Mg content <1%. The Dolomitic lime should have a Ca content of 20%–24% and a Mg content of 10%–14%. The recommended amount of lime is derived from target values for the Calcium percentage of base cations, which relates to pH—the rates are affected by cation exchange capacity, which is mainly determined by clay content in the soil. Recommended amounts are given as kg product per hectare.

The spatial variation patterns in the measured soil properties are not very useful for a farmer until translated to management advice. We therefore used the *Daktari-wa-Udongo* service of CropNuts Ltd. (Nairobi, Kenya) to derive management recommendations from soil data. A recommendation consists of a five-level classification (very low, low, optimal, high, and very high) of each soil property and suggested management option. If, for example, pH is low, liming is suggested as a first treatment before fertilizers are recommended, and the type of lime recommended is determined by the Ca:Mg ratio. Sensor-based maps for Kathande are shown as an example (Figure 8). Note the similarities of the spatial variation pattern among the maps.

## 6. Discussion

### 6.1. Three Conditions for a Working Prediction Model

The increased use of prediction models from soil sensors and through digital soil mapping, and their potential use in practical management in agriculture requires that relevant quality parameters of the outputs are reported. For a predictive model to be useful in practice, it needs to fulfill the following criteria:

1. Be accurate
2. Be applicable in larger areas, e.g., a county (spatially robust)
3. Be consistent over time (temporally robust)

The first criterion should be self-evident. There is no use of a model that does not give accurate predictions. But what is accurate and what is not? The mean error (ME) of a model may be very low. That means there is no considerable bias. At the same time, the mean absolute error (MAE), or the similar root mean squared error, RMSE, can be high. These values will tell how large the absolute error is on average, which is often more useful than just the ME. As described by Brus et al. [27], it is even more informative to look at the entire error distribution, or quantiles thereof. The accuracy of a model can be given with prediction limits, i.e., limits within which a specified percentage (often 95%) of the predictions are expected. It is not common to present uncertainty with prediction limits. An example is given by Odgers et al. [28], who present 5% and 95% prediction limits of their pH map. Another example (pH mapping and risk assessment of liming) is given by Gebbers et al. [29].

An important note in this context is that one should be careful before taking a sensor-based prediction model to use in practice. Government representatives in Embu County have shared their experience with us that inaccurate soil testing results from mobile labs have severely harmed the farmers’ trust in laboratory analyses in some areas. As it is very difficult for a farmer to judge the predictive power of a soil sensing technique, it is up to the service providers to judge whether a method is good enough to be taken to the market. We discourage uncritical use of proximal sensors for soil assessment. They are not multi-purpose tools that can predict all properties of the soil in the single push of a button, but they are useful for some tasks, and that is what this work has aimed to explore. Irrespective of whether you use a public map or in situ sensor measurements, be sure to validate the method for the extent and resolution of your intended use.

The second criterion should be self-evident too. Smallholder farms are often <1 hectares, so there is not much use of models that require soil samples for calibration at each farm. We have demonstrated (Figure 6) that the validation design is important if one wishes to investigate model performance under this precondition. If you want to know how well a method works on sites other than where the calibration samples were taken, be sure to design your validation strategy accordingly. A random cross-validation, where samples from the same site can be included in both the calibration and in the validation dataset is not good enough.

The third criterion was not possible to test in the present study and has therefore been neglected in this first screening. It is of importance to examine this in further tests of the models that have passed this first screening. A model that is calibrated for dry conditions but does not work after rainfall may not be useful in practice.

Additional aspects to keep in mind when interpreting the results of the present study are that (1) the accuracy of the reference method has not been taken into account and (2) differences in the sampling support/measurement footprint have been neglected. Any discrepancies can cause less high-quality models.

### 6.2. Geochemical Relevance of Prediction Models

What the present results show is simply that it was possible to calibrate robust and accurate models for the macronutrients N, K, Mg, Ca, and S, the micronutrients Zn, Mn, and Fe, and some other properties: TC, CEC, and exchangeable Al. This conclusion is based entirely on statistical grounds. Here we wish to discuss the geochemical relevance of the models that passed the two steps of screening. Are there possible cause–effect relationships or are the models simply working because of chance covariation among the response and the predictors?

Soil organic carbon and soil color has been demonstrated to be well correlated in several studies [11,12,30] and the optical sensor, used in this study, was designed specifically for mapping of TC content [17]. In this study, the best prediction model for TC was based on R, as measured with the optical sensor, and the contents of Sr and Zr, measured with the PXRF (i.e., no color variables). It may be worth mentioning here that, in this study, TC predictions based solely on the optical sensor did not perform as good as the combined PXRF optic mapper calibration (MAE = 1.8% TC for the leave-one-site-out calibration of the optical sensor alone, compared to MAE = 1.0% TC when the two sensors were combined).

### 6.3. Soil Texture Could Not Be Modeled with Any Satisfying Accuracy

When it comes to soil texture, we had expected working prediction models based on the current set of sensors. This hypothesis was based on the facts that (1) the texture of a soil affects the color of the soil and that is measured both by the optical sensor and the soil color app; (2) soil texture often affects ECa [15], which is measured by the EMI sensor; and (3) in some geological environments, the clay content has been demonstrated to have a strong linear relationship to the content of Rb in the soil [10,31], and that is one of the elements measured by the PXRF. Still, in the present study, no prediction model passed both the operational screening and the correlation screening, probably because the variation within sites was very modest. It may also be due to a different mineralogy compared to the soils investigated in the abovementioned studies. Iron oxide particles, for example, do not have the same properties as clay mineral particles, even though they may be of clay size. Kaolinite, which is a common clay mineral in weathered tropical soils, is also different from the 2:1-type clay minerals, which dominate the younger soils.

### 6.4. The EMI Sensor Is Good for Mapping of General Variation Patterns

Because several soil properties—dynamic properties, such as soil water content, salinity, temperature, and bulk density, and inherent soil properties, such as texture—which both impose an impact on ECa, the relationships between ECa and any individual soil property measured in the lab are often site-specific and the prospect of calibrating general prediction models valid for a larger area was therefore poor. In addition, the magnitude of variation within the individual fields was relatively small in this study (mean ± standard deviation for the shallow ECa values [mS·m^−1^] were: Irangi: 23 ± 3; Kathande: 7 ± 2; EUC: 15 ± 2; and Rwika: 14 ± 2). It was therefore not surprising that that the EMI sensor variables were not well correlated to any of the other variables in the study. There is also a discrepancy between the measurement depths of the EMI sensor and the depth of the soil samples, which may be (part of) the explanation to why there was no statistically significant correlation between the soil properties measured with the EMI sensor and the soil properties determined in the laboratory. All in all, this suggests that such instruments are better suited for mapping general variation patterns of a farm or a watershed, rather than for prediction of absolute values. The same conclusion has been drawn earlier by Piikki et al. [32].

### 6.5. Spatial Variation of Management Advice 

The present study showed that, although the investigated fields were small (≤1 ha), there was considerable within-site variation in the recommended amounts of inputs to apply (see example in Figure 8). With proximal sensors, it is possible to do numerous measurements to outline management zones within fields or farms [33,34,35], something that for economic reasons is most often not possible if relying solely on laboratory analyses. Using management zones is a simple version of precision agriculture.

There was also a substantial variation in management recommendations between sites, not only due to the fact that the advice was derived for different crops and yields. In the two fields at highest altitude (Irangi and Kathande), no fertilization was recommended until the soil pH has been corrected. That advice is probably general for a larger region around these sites, which indicates a need for soil information at multiple scales. Regional maps and local samples complement each other.

## 7. Conclusions

Our overall conclusion is that proximal sensors can be used for assessment of some, but not all, soil properties, and a well-designed validation is necessary before bringing any method out for use in practice:

1. The sensor-based models for N, K, Mg, Ca, S, Zn, Mn, Fe, TC, and CEC passed all the screening steps. In further tests, it is important to investigate the consistency of calibrations over time, since that has not yet been done. The PXRF and the optical sensor were the two sensors that were most often included in the functional, accurate, and robust models. Data from the soil color app proved useful in a few prediction models, while data from the EMI sensor were not included in any of the models passing the screening thresholds. We suggest that this instrument is best used for mapping general variation patterns, rather than predicting absolute values.
2. The leave-one-site-out validation method could identify spatially robust models, but the random three-fold validation could not.
3. We investigated how the variation in soil properties within and between sites translated to management advice given to farmers. This exercise revealed considerable variation in recommended management both within individual fields and within the region. We suggest using a combination of regional soil maps and local measurements to optimize management of small farms and thereby potentially increase yields. Management zones may be worth considering in heterogeneous fields.

## Figures and Tables

**Table 1:** Soil data (0–20 cm) for the studied sites. Mean ± standard deviation for all variables except CEC-clay; for CEC-clay the value for the deepest horizon in two soil profiles at each site is presented. Please note that any Fe oxides are in the clay fraction. EUC = Embu University College, TC = total carbon, N = nitrogen, and CEC = cation exchange capacity, Exch. Al = exchangeable Al.

Site	Irangi *n* = 36	Kathande *n* = 34	EUC *n* = 32	Rwika *n* = 43
**Texture**				
clay [%]	38 ± 5	42 ± 6	59 ± 5	39 ± 3
silt [%]	19 ± 2	17 ± 2	20 ± 3	27 ± 4
sand [%]	43 ± 5	40 ± 5	21 ± 4	35 ± 2
**Organic Matter**				
TC [%]	7.7 ± 1.3	5.8 ± 1.4	3.4 ± 0.7	2.2 ± 0.3
N [%]	0.6 ± 0.1	0.5 ± 0.1	0.3 ± 0.1	0.2 ± 0.0
**Easily Soluble Macronutrients**				
Ca [mg·kg^−1^]	59 ± 31	320 ± 150	1740 ± 675	1134 ± 384
K [mg·kg^−1^]	139 ± 45	158 ± 97	443 ± 217	528 ± 148
Mg [mg·kg^−1^]	26 ± 9	40 ± 17	342 ± 98	426 ± 91
P [mg·kg^−1^]	5 ± 2	21 ± 19	14 ± 9	3 ± 1
S [mg·kg^−1^]	38 ± 6	46 ± 15	12 ± 7	13 ± 5
**Easily Soluble Micronutrients**				
B [mg·kg^−1^]	0.5 ± 0.1	0.6 ± 0.8	1.3 ± 0.6	0.8 ± 0.3
Cu [mg·kg^−1^]	0.4 ± 0.1	14.4 ± 16	0.9 ± 1.4	1.1 ± 0.2
Fe [mg·kg^−1^]	151 ± 29	204 ± 55	76 ± 11	56 ± 11
Mn [mg·kg^−1^]	20 ± 8	32 ± 15	286 ± 33	175 ± 38
Zn [mg·kg^−1^]	2 ± 2	2 ± 2	17 ± 9	2 ± 1
**Other**				
pH	4.6 ± 0.2	4.2 ± 0.3	5.7 ± 0.5	6.1 ± 0.4
CEC [cmol_c_·kg^−1^]	2.6 ± 0.8	7.8 ± 2.9	18.2 ± 3.8	13.2 ± 2.4
CEC-clay [cmol_c_·kg^−1^]	4.1/3.4	3.7/1.9	11.6/16.7	7.4/5.6
Exch. Al [g·kg^−1^]	2.4 ± 0.1	2.0 ± 0.1	1.4 ± 0.2	1.2 ± 0.1

**Table 2:** Correlation matrix for soil properties. Correlations were tested for each site individually. The numbers indicate the numbers of sites where the correlation coefficient (r) was statistically significant (*p* < 0.05; disregarding potential spatial autocorrelation). The sign of the r values ± indicates that there were both significant positive correlations and significant negative correlations between the two variables considered. TC = total carbon, TN = nitrogen, and CEC = cation exchange capacity.

	Clay	Sand	Silt	TC	TN	Ca	K	Mg	P	S	B	Cu	Fe	Mn	Zn	pH	CEC	Al
clay	---																	
sand	+3	---																
silt	+4	0	---															
TC	+2	+2	+3	---														
TN	+1	+1	+3	+4	---													
Ca	+2	+3	+1	+1	0	---												
K	+1	+1	−1	+1	+1	+1	---											
Mg	+1	0	+2	±2	+2	0	+2	---										
P	+2	+4	+1	±2	+2	+2	+1	+1	---									
S	+2	+1	+3	+2	+2	0	+1	+2	0	---								
B	+1	+1	0	0	+3	+2	+1	0	0	+1	---							
Cu	-4	-2	−2	±2	−1	−1	−1	−1	0	−1	0	---						
Fe	+2	+2	0	−1	−1	+1	+1	0	+1	0	0	-3	---					
Mn	+2	0	+2	+1	+2	−1	0	+1	0	+1	0	-4	−1	---				
Zn	+4	+3	+4	+2	+1	+2	+1	+1	+3	+2	0	-4	+2	+2	---			
pH	±3	±3	+2	±4	+3	−2	+1	+1	−2	+2	+2	±2	−1	+1	±3	---		
CEC	+4	+3	+4	+4	+4	0	+1	+3	+1	+2	+2	-2	0	+2	+4	±3	---	
Al	±2	+1	−2	−2	−2	+1	0	−1	+1	−2	0	±4	+3	−1	±2	−2	−2	---

**Table 3:** Pairwise correlations between sensor variables and lab variables. Sensor variables for which less than 70% of the observations were above the detection limit at any of the sites were omitted from this analysis. The correlations were tested site-by-site and judged at the confidence level *p* < 0.05. − = one or more sites with a significant negative correlation but no site with a significant positive correlation; + = one or more sites with a significant positive correlation but no site with a significant negative correlation; ± = at least one site with a significant negative correlation and at least one site with a significant positive correlation; the numbers indicate the number of sites with significant correlations. TC = total carbon; TN = total nitrogen; and CEC = cation exchange capacity. The red reflectance (R) value of the optic mapper was a 20 nm band centered on 660 nm and that the R value of the app was the broad red band of the mobile phone camera. Exch. Al = exchangeable Al.

Laboratory Analysis	Optic Sensor		Color app		PXRF									
	R	IR	R Field	R lab	Cr	Fe	K	Mn	Pb	Rb	Sr	Ti	Zn	Zr
**Texture**														
clay	0	−1	0	0	0	0	0	0	0	0	+1	0	0	0
sand	0	+1	−1	0	+2	−1	0	0	±	−1	−1	0	±	−1
silt	−1	−1	+1	0	0	−1	0	−1	0	0	0	0	0	±
**Organic Matter**														
TC	−2	+1	−1	±	−3	−3	+1	±	−2	−2	±	−3	−2	−3
TN	−1	+1	−1	±	−3	−3	+1	±	−2	−2	±	−2	−2	−3
**Easily Soluble Macronutrients**														
Ca	−2	−1	−1	−1	0	0	+3	+1	0	+1	+2	0	0	0
K	−1	0	−2	−2	−2	−3	+3	±	−1	0	+1	−1	−1	−3
Mg	−2	−2	−1	−2	−2	−1	+1	±	−2	±	+2	−3	−2	−1
P	0	+1	0	±	−2	−2	±	−2	−2	−2	±	−1	−2	−2
S	0	+1	0	0	−1	−2	−1	−1	−1	−1	±	−1	−1	−2
**Easily Soluble Micronutrients**														
B	−2	−2	−1	−1	−1	−1	0	±	−1	0	+3	−2	0	−2
Cu	+1	−1	0	0	0	0	0	0	0	0	+1	0	0	0
Fe	0	+1	−2	−2	±	−2	±	−2	−3	−2	+1	−2	-2	−3
Mn	+1	+1	−1	−1	+1	0	+1	+3	+1	0	+2	0	+1	0
Zn	−1	0	0	−1	0	0	0	0	+1	0	+1	0	+2	0
**Other**														
pH	0	−2	−1	−1	+1	+1	+3	+3	+1	+2	±	+1	+2	+1
CEC	−2	−1	−1	−2	−1	−3	+2	±	−1	−1	+2	−3	−1	−3
Exch. Al	+1	+2	0	0	0	−2	0	−1	−1	0	−2	0	−1	−2

**Table 4:** Predictors included in at least one of the five best models for each response variable are marked 1, as judged by the sum of the ranks of r^2^, E, and MAE for the leave-one-site-out validations. Predictor variables that were not included among the best are denoted 0. Prediction models with an E-value < 0.5 were considered non-functional and were thus omitted from the present summary, as well as from any further analyses. The stars (*) indicate that the predictor was included in the best model (the one of the five best models that had the highest number of measurements above the detection limit), for which a more detailed presentation of the validation results is found in Figure 5. CEC = cation exchange capacity; R = red, IR = infrared; TC = total carbon; N = nitrogen; Exch. Al = exchangeable Al; --- = no working prediction model for this soil property. The red reflectance (R) value of the optic mapper was a 20 nm band centered on 660 nm and the R value of the app was the broad red band of the mobile phone camera.

Laboratory Analysis	Optic Sensor			Color app		PXRF									
	R	IR	R/IR	R Field	R lab	Cr	Fe	K	Mn	Pb	Rb	Sr	Ti	Zn	Zr
**Texture**															
clay	---	---	---	---	---	---	---	---	---	---	---	---	---	---	---
sand	---	---	---	---	---	---	---	---	---	---	---	---	---	---	---
silt	---	---	---	---	---	---	---	---	---	---	---	---	---	---	---
**Organic Matter**															
TC	1 *	1	0	1	0	1	0	0	0	0	0	1 *	1	0	1 *
TN	1	0	0	0	0	1	0	1 *	0	1	1*	1	1	0	1 *
**Easily Soluble Macronutrients**															
Ca	1 *	1	0	0	0	1	0	0	0	0	0	1 *	0	0	1
K	1 *	0	1	1	0	1	1 *	1 *	0	0	1	1	0	0	0
Mg	1	0	1 *	1	1	0	0	1 *	0	1	0	0	0	0	0
P	---	---	---	---	---	---	---	---	---	---	---	---	---	---	---
S	1	0	1 *	1	0	0	0	1	0	0	0	0	1	0	0
**Easily Soluble Micronutrients**															
B	---	---	---	---	---	---	---	---	---	---	---	---	---	---	---
Cu	---	---	---	---	---	---	---	---	---	---	---	---	---	---	---
Fe	1 *	1 *	1	1	0	0	0	1	0	0	0	0	1	0	0
Mn	0	0	0	0	1	1	1	0	1	0	0	1 *	0	1 *	1
Zn	1 *	1	1	0	0	0	0	1	0	0	0	1 *	0	1 *	0
**Other**															
pH	1 *	1	1 *	1	0	1	0	1 *	0	0	0	0	0	0	0
CEC	0	0	1 *	1	1	1	0	1	0	1	0	1 *	0	0	0
Exch. Al	0	0	0	1	0	0	1 *	0	1	0	1 *	1 *	1	1	1

## Supplementary Materials

The following are available online at http://www.mdpi.com/1424-8220/16/11/1950/s1, Table S1: Data on soil profiles at Irangi. TC = total carbon, N = nitrogen and CEC = cation exchange capacity, Base sat. = degree of base saturation, Table S2: Data on soil profiles at Kathande. TC = total carbon, N = nitrogen and CEC = cation exchange capacity, Base sat. = degree of base saturation, Table S3: Data on soil profiles at Embu University Campus (EUC). TC = total carbon, N = nitrogen and CEC = cation exchange capacity, Base sat. = degree of base saturation. Table S4: Data on soil profiles at Rwika. TC = total carbon, N = nitrogen and CEC = cation exchange capacity, Base sat. = degree of base saturation.
