# Supplementary material for: Performance Evaluation of Proximal Sensors for Soil Assessment in Smallholder Farms in Embu County, Kenya
Source: Sensors (Basel). 2016 Nov 19;16(11):1950. doi: 10.3390/s16111950 (PMC5134609; doi:10.3390/s16111950)
Supplement: Supplementary file 1 [file sensors-16-01950-s001.pdf]

Supplementary Materials: Performance Evaluation of Proximal Sensors for Soil Assessment in Smallholder Farms in Embu County, Kenya

Kristin Piikki, MatsSöderström, Jan Eriksson, Jamleck Muturi John, Patrick Ireri Muthee, Johanna Wetterlind and Eric Lund

Table S1. Data on soil profiles at Irangi. TC = total carbon, N = nitrogen and CEC = cation exchange capacity, Base sat. = degree of base saturation.

Depth (cm)	Particle Size Distri-Bution (%)			Oganic Matter (%)		pH	Exchangeable Cations ($cmol_c \cdot kg^{-1}$)						Base sat.
	Sand	Silt	Clay	TC	N		K	Ca	Mg	Na	Al	CEC	(%)
						Soil Profile 1							
0–6	-	-	-	15.6	0.99	4.1	0.6	1.0	0.9	0.2	9.8	9.6	28
6–14	52	16	32	9.7	0.73	4.9	0.3	0.3	0.2	0.2	3.4	2.3	44
14–25	36	20	44	4.8	0.40	4.7	0.3	0.6	0.1	0.2	2.9	3.0	41
25–35	40	16	44	3.7	0.30	4.8	0.3	0.1	0.1	0.2	2.9	1.5	43
35–50	38	18	44	3.3	0.27	4.6	0.2	0.7	0.2	0.2	2.4	3.5	37
50–70	36	16	48	2.9	0.24	4.3	0.1	0.3	0.1	0.2	2.1	2.2	31
70–90	36	14	50	2.9	0.23	4.6	0.1	0.3	0.1	0.3	1.4	2.1	39
						Soil Profile 2							
0–3	-	-	-	21.7	1.24	4.3	0.6	6.4	3.6	0.2	5.2	34.7	31
3–13	38	16	46	2.9	0.26	4.5	0.3	0.3	0.2	0.2	2.2	2.6	33
13–20	42	18	40	5.6	0.46	4.6	0.1	0.3	0.1	0.1	4.1	1.7	36
20–40	42	16	42	3.9	0.33	4.4	0.1	0.4	0.1	0.2	3.2	2.6	38
40–60	36	14	50	2.3	0.21	4.4	0.1	0.4	0.1	0.3	1.4	2.8	35
60–80	36	16	48	2.6	0.23	4.6	0.1	0.3	0.1	0.2	0.8	1.7	39
80–100	38	14	48	2.5	0.22	4.7	0.1	0.3	0.1	0.2	0.9	1.6	40

Table S2. Data on soil profiles at Kathande. TC = total carbon, N = nitrogen and CEC = cation exchange capacity, Base sat. = degree of base saturation.

Depth (cm)	Particle Size Distri-Bution (%)			Oganic Matter (%)		pH	Exchangeable Cations ($cmol_c \cdot kg^{-1}$)						Base sat.
	Sand	Silt	Clay	TC	N		K	Ca	Mg	Na	Al	CEC	(%)
						Soil Profile 1							
0–10	46	16	38	7.9	0.71	4.2	0.3	2.7	0.6	0.2	6.0	12.6	29
10–20	42	22	36	4.3	0.39	4.5	0.1	1.7	0.3	0.0	5.1	5.9	36
20–30	40	16	44	3.6	0.34	4.6	0.1	1.2	0.2	0.1	4.2	4.4	37
30–40	34	16	50	4.5	0.41	4.5	0.1	1.0	0.2	0.0	4.1	3.8	37
40–60	32	10	58	2.3	0.25	4.5	0.1	0.8	0.2	0.2	4.6	3.5	35
60–80	32	10	58	2.0	0.22	4.3	0.1	0.5	0.1	0.1	4.2	2.4	32
80–100	26	6	68	1.8	0.20	4.3	0.1	0.5	0.1	0.2	3.6	2.5	33
						Soil Profile 2							
0–5	60	14	26	11.3	0.97	4.1	0.4	2.9	0.4	0.1	7.0	14.4	27
5–15	30	14	56	3.1	0.33	4.3	0.3	0.6	0.2	0.2	5.1	4.0	29
15–25	24	8	68	2.3	0.23	4.3	0.2	0.4	0.1	0.1	4.8	2.5	32
25–35	26	8	66	2.2	0.22	4.2	0.1	0.3	0.1	0.0	5.0	1.7	31
35–45	24	10	66	2.0	0.21	4.2	0.1	0.3	0.1	0.1	4.6	2.0	30
45–60	36	18	46	4.5	0.39	4.2	0.1	0.3	0.1	0.1	5.2	1.8	29
60–80	24	8	68	-	-	4.4	0.1	0.3	0.1	0.0	-	1.3	33

Table S3. Data on soil profiles at Embu University Campus (EUC). TC = total carbon, N = nitrogen and CEC = cation exchange capacity, Base sat. = degree of base saturation.

Depth (cm)	Particle Size Distri-Bution (%)			Oganic Matter (%)		pH	Exchangeable Cations (cmol$_c$·kg^{-1})						Base sat.
	Sand	Silt	Clay	TC	N		K	Ca	Mg	Na	Al	CEC	(%)
**Soil Profile 1**													
0–10	29	20	51	3.6	0.36	5.1	1.2	3.8	1.5	0.2	2.1	13.5	50
10–20	23	20	57	2.7	0.28	5.1	0.8	3.5	1.3	0.1	1.6	11.8	49
20–30	21	14	65	2.1	0.23	5.1	0.6	3.0	1.3	0.1	1.9	9.8	51
30–40	19	12	69	2.0	0.22	5.0	0.4	2.7	1.3	0.1	1.8	10.1	46
40–60	17	10	73	1.8	0.20	4.9	0.3	2.8	1.4	0.2	1.5	10.4	45
60–80	13	16	71	1.3	0.18	5.6	0.2	3.7	2.4	0.1	0.2	9.9	65
80–100	11	16	73	1.1	0.16	5.9	0.1	3.0	2.9	0.3	0.0	8.4	74
**Soil Profile 2**													
0–10	19	18	63	4.0	0.35	5.8	1.0	7.8	4.1	0.2	0.1	18.4	71
10–20	15	20	65	2.5	0.25	5.4	0.6	4.4	3.0	0.2	0.6	13.7	59
20–30	13	16	71	1.9	0.21	5.1	0.4	2.9	2.5	0.2	2.2	11.9	51
30–40	11	12	77	1.7	0.18	5.1	0.3	2.9	2.6	0.2	1.4	12.3	49
40–50	11	12	77	1.4	0.16	5.2	0.2	2.8	2.8	0.1	1.0	11.5	52
50–75	9	8	83	1.1	0.14	5.3	0.2	2.4	2.7	0.2	0.6	9.8	56
75–100	9	8	83	0.8	0.13	4.8	0.3	2.2	3.1	0.3	0.3	13.9	41

Table S4. Data on soil profiles at Rwika. TC = total carbon, N = nitrogen and CEC = cation exchange capacity, Base sat. = degree of base saturation.

Depth (cm)	Particle Size Distri-Bution (%)			Oganic Matter (%)		pH	Exchangeable Cations (cmol$_c$·kg^{-1})						Base sat.
	Sand	Silt	Clay	TC	N		K	Ca	Mg	Na	Al	CEC	(%)
**Soil Profile 1**													
0–10	34	36	30	2.4	0.22	6.3	1.7	6.6	4.5	0.0	0.2	15.4	83
10–20	32	24	44	1.6	0.16	5.6	1.2	2.6	2.2	0.0	0.4	9.3	65
20–40	32	16	52	1.0	0.12	4.9	0.9	1.0	1.1	0.1	2.0	6.6	46
40–60	34	12	54	0.9	0.10	4.9	0.6	0.3	0.6	0.0	3.0	3.2	46
60–80	32	16	52	0.8	0.09	4.9	0.6	0.4	0.8	0.1	2.3	4.2	45
80–100	30	20	50	0.7	0.09	5.0	0.4	0.4	0.9	0.1	2.6	3.7	47
**Soil Profile 2**													
0–10	32	28	40	2.1	0.19	6.2	1.3	5.5	4.1	0.1	0.0	13.4	82
10–20	30	20	50	1.5	0.14	5.0	0.5	2.0	2.3	0.0	1.0	10.0	48
20–40	36	20	44	1.2	0.12	4.5	0.3	0.6	0.8	0.1	3.2	4.6	36
40–60	38	24	38	1.1	0.11	4.8	0.1	0.3	0.7	0.0	2.9	2.6	43
60–80	36	28	36	0.9	0.11	5.0	0.1	0.3	0.6	0.0	2.5	1.9	46
80–100	30	32	38	0.8	0.10	5.0	0.1	0.3	0.6	0.1	2.2	2.1	48
